# PCR-based rapid genotyping of *Stenotrophomonas maltophilia *isolates

**DOI:** 10.1186/1471-2180-8-202

**Published:** 2008-11-24

**Authors:** Emanuela Roscetto, Francesco Rocco, M Stella Carlomagno, Mariassunta Casalino, Bianca Colonna, Raffaele Zarrilli, Pier Paolo Di Nocera

**Affiliations:** 1 Dipartimento di Biologia e Patologia Cellulare e Molecolare, Università Federico II, Via S. Pansini 5, 80131 Napoli, Italy; 2 Dipartimento di Biologia, Università Roma TRE, Viale Marconi 446 Roma, Italy; 3 Istituto Pasteur-Fondazione Cenci Bolognetti, Dipartimento di Biologia Cellulare e dello Sviluppo, Università Roma La Sapienza, Via dei Sardi 70 Roma, Italy; 4 Dipartimento di Scienze Mediche Preventive, Sezione di Igiene, Università Federico II, Via S. Pansini 5, 80131 Napoli, Italy

## Abstract

**Background:**

All bacterial genomes contain repetitive sequences which are members of specific DNA families. Such repeats may occur as single units, or found clustered in multiple copies in a head-to-tail configuration at specific loci. The number of clustered units per locus is a strain-defining parameter. Assessing the length variability of clusters of repeats is a versatile typing methodology known as multilocus variable number of tandem repeat analysis (MLVA).

**Results:**

*Stenotrophomonas maltophilia *is an environmental bacterium increasingly involved in nosocomial infections and resistant to most antibiotics. The availability of the whole DNA sequence of the *S. maltophilia *strain K279a allowed us to set up fast and accurate PCR-based diagnostic protocols based on the measurement of length variations of *loci *carrying a variable number of short palindromic repeats marking the *S. maltophilia *genome. On the basis of the amplimers size, it was possible to deduce the number of repeats present at 12 different *loci *in a collection of *S. maltophilia *isolates, and therefore label each of them with a digit. PCR-negative regions were labelled 0. Co-amplification of two pairs of *loci *provided a 4-digit code sufficient for immediate subtyping. By increasing the number of *loci *analyzed, it should be possible to assign a more specific digit profile to isolates. In general, MLVA data match genotyping data obtained by PFGE (pulsed-field gel electrophoresis). However, some isolates exhibiting the same PCR profiles at all *loci *display distinct PFGE patterns.

**Conclusion:**

The utilization of the present protocol allows to type several *S. maltophilia *isolates in hours. The results are immediately interpretable without the need for sophisticated softwares. The data can be easily reproducible, and compared among different laboratories.

## Background

After years of debate regarding its appropriate taxonomic position, the nonfermentative, gram-negative bacillus previously known as *Pseudomonas maltophilia *or *Xanthomonas maltophilia*, has been definitively classified as *Stenotrophomonas maltophilia *[1]. This species is found in a wide variety of environments, and has been isolated from different sources, including water, sewage, soil and plant rhizosphere environments [2]. *S. maltophilia *is increasingly prevalent in hospitals, and is often isolated in hospitalized patients, as well as in cystic fibrosis (CF), burn, and immunosuppressed patients. The presence of *S. maltophilia *in CF patients is not associated with a worse clinical outcome. However, the organism contributes to chronic airway inflammation [3]. Moreover, in mixed infection formed in the CF lungs, *S. maltophilia *has been shown to influence the architecture of *Pseudomonas aeruginosa *biofilms by producing a diffusable signal factor [4].

*S. maltophilia *isolates exhibit high genetic diversity. Genotypic profiles have been determined by a variety of methods, including AFLP (amplified fragment length polymorphism) fingerprinting [5], RFLP (restriction fragment length polymorphism) analysis of the gyrase B gene [6] or the intergenic region between *sme*D and *sme*T genes [7], ERIC-PCR [8], and PFGE (pulsed-field gel electrophoresis) analysis of *Xba *I genomic digests [8-11].

Genome-wide analyses showed that in many bacterial genomes short DNA segments are amplified in tandem at specific chromosomal *loci *. Changes in the number of repeats among isolates can be monitored by PCR, and MLVA (Multi *locus *variable number of tandem repeat analysis) surveys are widely used for subtyping purposes [12-19].

The sequence of the genome of the *S. maltophilia *strain K279a has been completed [20]. Genome inspection allowed us to set up a simple, fast and accurate PCR-based diagnostic protocol which relies on the measurement of length heterogeneity of specific intergenic regions of the *S. maltophilia *genome.

The present protocol allows typing several *S. maltophilia *isolates in hours. The assignment of a digit code to each isolate could be used to easily compare data among different laboratories.

## Results

### Specific sequence repeats punctuate the genome of *S. maltophilia*

The whole DNA sequence of the *S. maltophilia *strain K279a has been determined [20]. The genome is 4,851,126 bp in length, and has an average G+C content of 66.3%. We found that the K279a chromosome hosts an abundant family of small, palindromic repeats fitting the consensus GTAGTGCCGGCCGCTGGCCGGCA (complementary residues are underlined) that we called SMAG (for Stenotrophomonas MAltophilia GTAG) because they carry the tetranucleotide GTAG at one terminus, similarly to small repetitive extragenic palindromic sequences (REPs) identified in the genomes of *Escherichia coli *and other microrganisms [21]. SMAGs make up approximately 0.5% of the K279a genome, and are spread throughout the chromosome either as single units, or in pairs, separated by 5–80 bp long spacers. The size of the SMAG family allows to hypothesize that some of these repeats may function as regulatory signals either at the DNA or the RNA level, as shown for REPs [21].

### SMAGs and the PCR-based genotyping of *S. maltophilia *isolates

**Figure 1:**
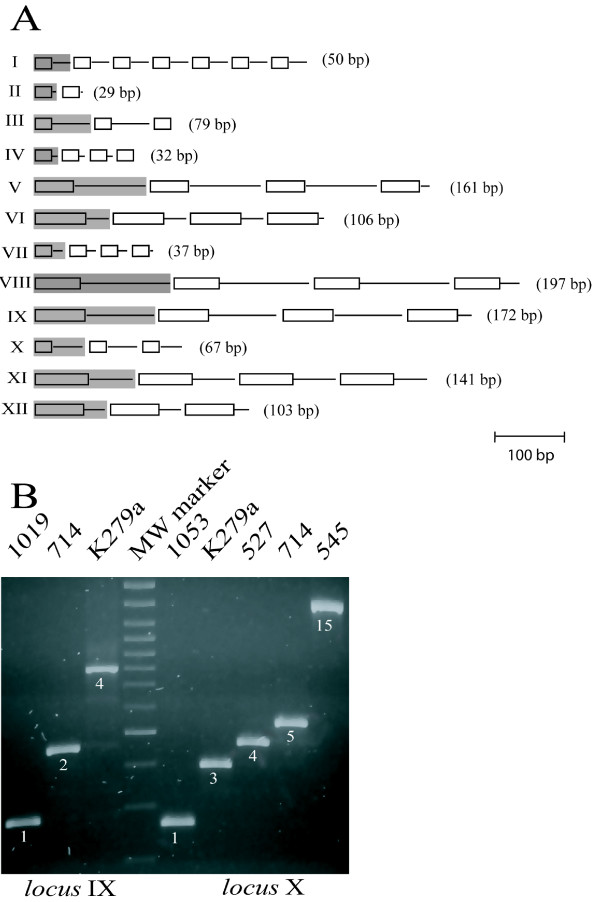
**A) Organization of the repetitive SMAG-positive *loci *in the genome of the *S. maltophilia *K279a strain**. The structure of *loci *I to XII is diagramatically shown. SMAG sequences are represented as boxes. Repeat units at each locus are highlighted and their size in bp is indicated. B) Allelic variations at SMAG^+ ^*loci*. Amplimers spanning the *loci *IX and X derived from the DNA of the indicated *S. maltophilia *strains were electrophoresed on a 2% agarose gel. Numbers below bands mark the number of repeat units within each amplimer. The 100 bp ladder was used as molecular weight (MW) DNA marker.

In the K279a chromosome, monomeric and dimeric SMAGs are reiterated in tandem at multiple chromosomal *loci*, along with tracts of variable length of flanking DNA. We exploited the occurrence of SMAG arrays to set up PCR-based typing protocols, and focused our attention on 12 such *loci*, labelled I to XII in accord to their location on the K279a chromosome (Table 2). No rule in the pattern of amplification of SMAG sequences at the different *loci *could be discerned (Fig. 1A). Thus, region I features 50 bp long repeats, resulting from the duplication of a monomeric 24 bp long SMAG along with 26 bp of flanking DNA. In contrast, region XII features repeats which are 103 bp in length, and results from the duplication of a dimeric 72 bp SMAG and 31 bp of flanking DNA. In pilot experiments, regions IX and X were amplified by PCR from the DNA of the control strain K279a and five different *S. maltophilia *isolates. As shown in Fig. 1B, the size of the SMAG-positive regions varies, and this correlates with changes in the number of repeating units as confirmed by sequence analysis. Isolates could thus be marked by a digit corresponding to the number of SMAG repetitions present at a given *locus *(Fig. 1B). Prompted by these results, we monitored the twelve SMAG-positive *loci *by PCR. Analyses were carried out on DNAs derived from 38 *S. maltophilia *strains, including the K279a strain, isolated from different sources (Table 1). On the basis of the amplimers size, it had been possible to deduce the number of repeats present at the *loci *in the various isolates, and therefore label each of them with a digit (Table 3). In some instances, we could not detect an amplification product for one or more *loci *in different isolates. PCR-negative regions were labelled 0. To confirm our findings, alternative primers were used for some of these regions, but no reliable amplification product could be detected. The lack of amplification may reflect either an extensive polymorphism or deletions occurred in the regions analyzed.

**Table 1:** Source and origin of the *S. maltophilia *strains analyzed in this study

strain name	source	location	reference
92	bronchial aspirate (ICU)	UFH, Naples	[10]
262	bronchial aspirate (CF)	UFH, Naples	[10]
527	pharyngeal swab (H)	UFH, Naples	[10]
528	bronchial aspirate (ICU)	UFH, Naples	[10]
545	bronchial aspirate (CF)	UFH, Naples	[10]
549	bronchial aspirate (ICU)	UFH, Naples	[10]
571	bronchial aspirate (ICU)	UFH, Naples	[10]
598	pharyngeal swab (H)	UFH, Naples	[10]
616	pharyngeal swab (H)	UFH, Naples	[10]
707	bronchial aspirate (CF)	UFH, Naples	[10]
714	pharyngeal swab (H)	UFH, Naples	[10]
915	bronchial aspirate (ICU)	UFH, Naples	[10]
916	bronchial aspirate (ICU)	UFH, Naples	[10]
1019	bronchial aspirate (ICU)	UFH, Naples	[10]
1029	bronchial aspirate (ICU)	UFH, Naples	[10]
1039	bronchial aspirate (ICU)	UFH, Naples	[10]
1053	urine (ICU)	UFH, Naples	[10]
1054	bronchial aspirate (ICU)	UFH, Naples	[10]
OBGTC3	pharyngeal swab (CF)	BGH, Rome	this study
OBGTC9	bronchial aspirate (CF)	BGH, Rome	[28]
OBGTC10	bronchial aspirate (CF)	BGH, Rome	[28]
OBGTC13	bronchial aspirate (CF)	BGH, Rome	this study
OBGTC16	pharyngeal swab (CF)	BGH, Rome	[28]
OBGTC20	bronchial aspirate (CF)	BGH, Rome	[28]
OBGTC22	bronchial aspirate (CF)	BGH, Rome	this study
OBGTC23	bronchial aspirate (CF)	BGH, Rome	[28]
OBGTC26	pharyngeal swab (CF)	BGH, Rome	[28]
OBGTC28	bronchial aspirate (CF)	BGH, Rome	[28]
OBGTC29	bronchial aspirate (CF)	BGH, Rome	[28]
OBGTC30	bronchial aspirate (CF)	BGH, Rome	this study
OBGTC45	bronchial aspirate (CF)	BGH, Rome	this study
OBGTC75	bronchial aspirate (CF)	BGH, Rome	this study
STM2	emocolture (H)	BGH, Rome	this study
K279a	emocolture (C)	BOU, Bristol	[20]
LMG959	rice paddy	Japan	this study
LMG10879	rice paddy	Japan	this study
LMG10871	soil	Japan	this study
OBG N1	soil	BGH, Rome	this study

CF, Cystic Fibrosis; ICU, Intensive Care Unit; H, Haematology; C (Cancer); UFH, University Federico II Hospital; BGH, Bambino Gesù Hospital; BOU, Bristol Oncology Unit. LMG-labeled strains are from the Laboratorium voor Microbiologie Gent Culture Collection, Belgium.

**Table 2:** *Loci *analyzed by PCR in *S. maltophilia *strains

***locus***	**coordinates**	**PCR primers**	**Ta**
**I**	0078153–0078502	f [0077906] CACCGCCGAGTGCGATGCCGATCTT	69°C
		r [0078751] ACCCGACCGTGGACATGGACGTGCG	
			
**II**	0458218–0458270	f [0458122] GACGTGAAGTGGCTGCGCCTGAAGC	65°C
		r [0458343] CGTTCCAGCCACTGTACCGCCACCA	
			
**III**	0898001–0898182	f [0897802] GTGGTGGTGATCAAGCGCGGCAAGG	68°C
		r [0898549] GGCAGGTCGGCTGGATGGCGGTACT	
			
**IV**	1260576–1260695	f [1260393] CAGGAACGATGTGCGGGCAGTGACC	66°C
		r [1260806] CTGTCCGAAACACATGGCGTGGCAG	
			
**V**	1397805–1398345	f [1397652] TGATCGGCATCATCGTGGTCGGTAC	65°C
		r [1398470] GCGAGTACCTGAGCGAACTGGGGTG	
			
**VI**	2363422–2363811	f [2363314] CAGCATCATCAACAAGCACCATGGC	65°C
		r [2363972] TATCGCTTCCTGACCAAACCGTGGA	
			
**VII**	2877734–2877879	f [2877624] GCCGCTGGTCTGGCCGTTGATGATG	65°C
		r [2877987] GCTGGAGCTGCACCTGAGCGCCTGG	
			
**VIII**	2893713–2894397	f [2893497] TGGACCGCCACCGACTACCTGATGG	67°C
		r [2894486] CACCACCACCACCGAGGTCTACCCG	
			
**IX**	3626782–3627370	f [3626652] CGAGTACTTCACCCCGGTCAACGAG	65°C
		r [3627448] AGGGCCAGACCTTCGAGGAATTCAA	
			
**X**	3842432–3842620	f [3842338] TGGTGGTCAATGATGGGCAGCCGGA	66°C
		r [3842731] CCGCCACGATGACTGGTCTCAGCCG	
			
**XI**	4475729–4476245	f [4475549] CACAGGTCACACAGCGTGGTGTACG	65°C
		r [4476369] GGTTGTTCGGCATCGGTTTGATCAT	
			
**XII**	4528877–4529176	f [4528752] CGCCATCCAGCCGTCCTGTACTGCT	66°C
		r [4529342] GGCGGGTTGGGTGGGTACTACCTGG	

The coordinates of the *loci *on the genome of the K279a strain, the forward (f) and reverse (r) primers in the 5'-3' orientation, their 5' end position, and the annealing temperatures (Ta) are shown.

**Table 3:** PCR analysis of SMAG+ *loci *in *S. maltophilia *strains.

strains	*loci*												PCR Type
	II	V	I	VII	IX	III	VIII	VI	XII	XI	X	IV	
													
OBGTC28	**1**	**0**	**2**	**2**	1	2	1	1a	1	0	0	0	PT-1
LMG959	**1**	**0**	**2**	**2**	0	1	0	1c	2	0	0	0	
1054	**1**	**1**	**0**	**4**	1	1	0	1c	1	1b	0	0	PT-2
OBG N1	**1**	**1**	**0**	**3**	1	5	1	1a	1	0	0	0	PT-3
1029	**1**	**1**	**1**	**2**	1	1	0	1b	1	1b	0	0	PT-4
1039	**1**	**1**	**1**	**2**	1	1	0	1b	1	1b	1	2	
616	**1**	**1**	**2**	**2**	0	0	0	0	0	0	0	0	PT-5
707	**1**	**1**	**2**	**2**	1	0	0	8	0	0	0	0	
LMG10879	**1**	**1**	**3**	**2**	0	1	0	0	0	0	17	0	PT-6
OBGTC22	**2**	**0**	**0**	**0**	0	0	0	0	0	0	4	0	PT-7
LMG10871	**2**	**0**	**0**	**3**	0	0	1	2	0	0	0	0	PT-8
92	**2**	**0**	**4**	**2**	1	1	1	1a	1	2	0	2	PT-9
549	**2**	**1**	**0**	**2**	1	0	0	1c	1	0	0	0	PT-10
598	**2**	**1**	**0**	**2**	2	6	1	4	4	1a	4	2	
OBGTC23	**2**	**1**	**0**	**2**	1	2	1	1a	1	2	0	0	
STM2	**2**	**1**	**2**	**2**	1	1	1	2	1	1a	4	0	PT-11
OBGTC75	**2**	**1**	**3**	**1**	4	2	1	1b	1	4	0	2	PT-12
OBGTC13	**2**	**1**	**4**	**2**	1	2	1	1a	1	2	4	0	PT-13
OBGTC30	**2**	**1**	**4**	**2**	1	2	1	1a	0	0	0	0	
916	**2**	**3**	**3**	**2**	1	1	4	8	2	4	1	2	PT-14
1019	**2**	**3**	**3**	**2**	1	1	4	8	2	4	1	2	
1053	**2**	**3**	**3**	**2**	1	1	4	8	2	4	1	2	
527	**2**	**3**	**7**	**4**	4	1	3	1a	4	4	4	4	PT-15
OBGTC29	**2**	**4**	**7**	**1**	2	2	4	3	4	3	15	2	PT-16
714	**2**	**4**	**7**	**2**	2	3	4	6	2	4	5	3	PT-17
545	**2**	**4**	**7**	**3**	2	2	4	3	3a	3	15	2	PT-18
262	**2**	**4**	**7**	**4**	2	3	4	6	2	4	5	3	PT-19
915	**2**	**4**	**7**	**4**	2	3	4	1a	2	3	5	4	
K279a	**2**	**4**	**7**	**4**	4	3	4	4	3b	4	3	4	
OBGTC9	**2**	**4**	**7**	**4**	4	3	4	4	3b	4	3	4	
OBGTC10	**2**	**4**	**7**	**4**	4	3	4	4	3b	4	3	4	
OBGTC20	**2**	**4**	**7**	**4**	4	3	4	1a	4	4	3	2	
OBGTC26	**2**	**4**	**7**	**4**	2	1	4	6	2	4	5	4	
528	**2**	**5**	**3**	**3**	4	2	4	5	5	1a	5	4	PT-20
571	**2**	**5**	**3**	**3**	4	2	4	5	5	1a	5	4	
OBGTC16	**2**	**5**	**5**	**4**	5	3	3	7	3a	3	4	4	PT-21
OBGTC3	**2**	**5**	**10**	**2**	5	3	3	9	3a	3	4	3	PT-22
OBGTC45	**2**	**5**	**10**	**4**	5	0	3	9	3a	0	15	5	PT-23

The PCR products derived from the amplification of *loci *I to XII in the listed strains are labeled with the number of SMAG repeats present. The letters a, b and c denote length differences among amplimers assigned to the same class. The lack of amplification is labeled by zero. PCR types (PTs) are defined by the 4-digit code resulting from analyses of *loci *II, V, I and VII (bold numbers). Strains with the same PT are boxed.

A few PCR products, derived from the amplification of regions VII, XI and XII, were slightly different in length, and could not be assigned to a size class. In these instances, amplimers were assigned to the nearest size class, and marked with the letters a, b and c to denote size differences among them (Table 3). To clarify this issue, we determined the sequence of the PCR products derived by amplification of region VI in strains 915, 1029 and LMG959 (classified in Table 3 as 1a, 1b and 1c, respectively). In the control strain K279a, region VI contains four SMAG dimers. In the 915 strain, the amplified DNA (1a amplimer) was similar to the K279a interval, but only one SMAG dimer was present. In the LMG959 strain, the region amplified (1c amplimer) was 45 bp shorter, because the SMAG dimer was replaced by a SMAG monomer. In strain 1029, the size change of region VI (1b amplimer) was due to replacement of the SMAG dimer and 10 bp flanking sequence by a 66 bp palindromic element, that is a member of a distinct, less abundant family of GTAG^+ ^repeats in *S. maltophilia*. Thus, size variations of *loci *analyzed may correlate with recombinational events which replace SMAGs with members of the same family, or related DNA families.

In order to assess the stability of the SMAG-positive regions, the strains 528, 916 and 1039 were sub-cultured for 5 days, and the DNA extracted from single colonies of each strain was analyzed by PCR (data not shown). No changes in the pattern of amplification at *loci *III, IV, IX and XII were observed.

As shown in Table 3, the information derived from the survey of *loci *II, V, I and VII was sufficient to obtain a 4-digit code, that assigned the 38 DNAs analyzed to 23 different PTs (PCR Types). Some strains, such as 528 and 571, belong to the same PT type, and exhibited the same PCR profile at all the other *loci*. The same holds true for the three PT type 14 strains 916, 1019 and 1053, and for 3 out of 7 of the PT-19 strains. In contrast, strains 714 an 262 belong to different PTs (17 and 19, respectively), but, aside from differences in region VII, were identical at all *loci*.

**Figure 2:**
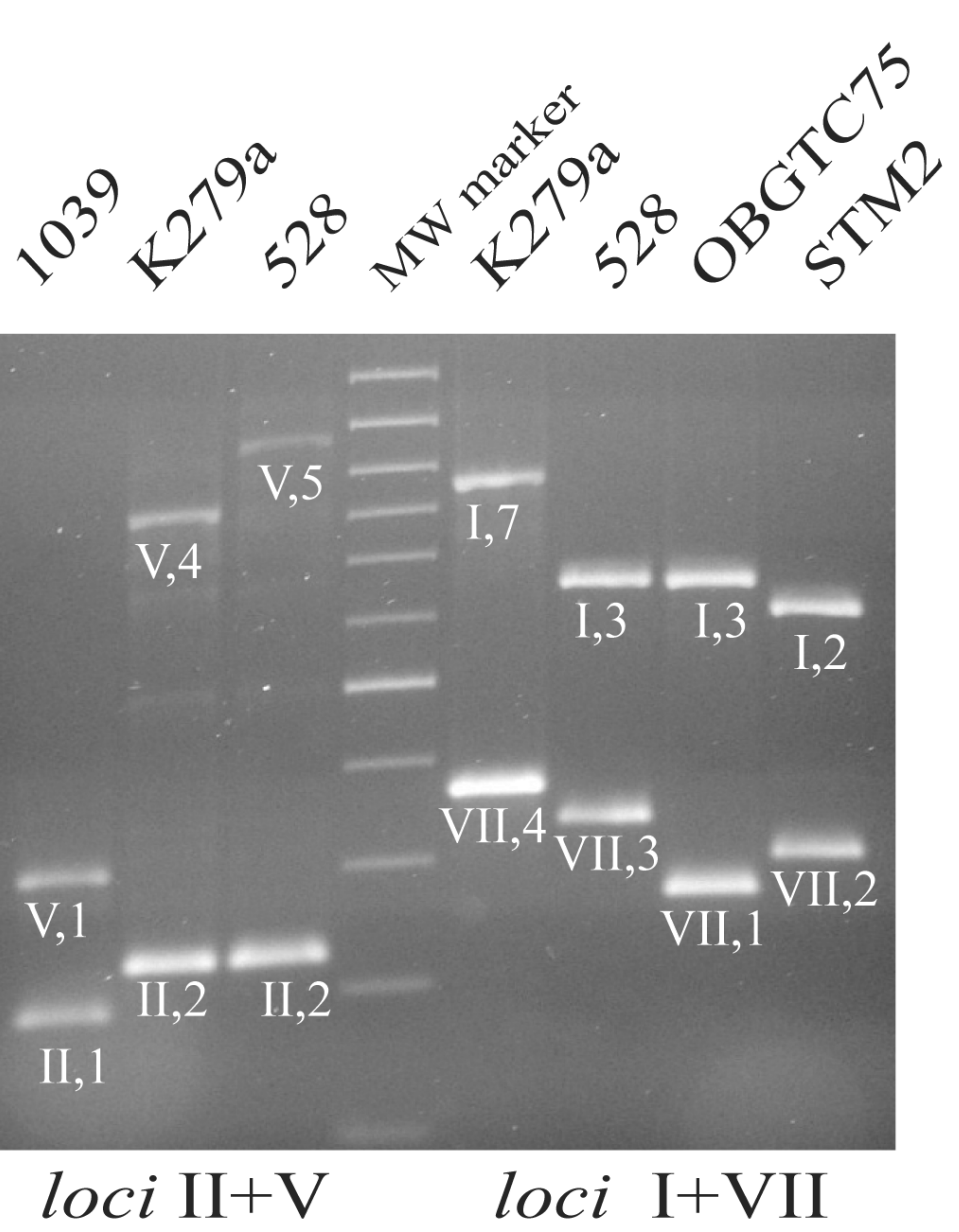
**Co-amplification of SMAG-positive *loci***. Amplimers deriving from the dual amplification of II and V, and I and VII *loci *from the DNA of the indicated strains were analyzed by elctrophoresis as in Fig. 1.

The finding that a comparatively relatively robust typing can be achieved by analysing only the four *loci *II, V, I and VII, highlighted in Table 3, is relevant, mostly in view of the fact that they can be co-amplified in pairs. In both instances, the amplimers corresponding to either *locus *can be easily distinguished because of their size range, allowing an immediate typing (Fig. 2).

### PFGE-typing of *S. maltophilia *isolates

**Figure 3:**
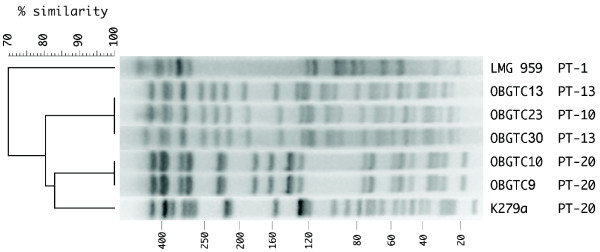
**PFGE profiles of *S. maltophilia *strains**. The DNA of the indicated isolates was restricted with *Xba*I. The digestion products were resolved on 1.2% agarose gels (see Methods). The percentage genetic similarity is shown above the dendrogram. Strain numbers and PT types are shown on the right of each PFGE profile. Numbers at the bottom refer to the size in kb of the MW DNA.

PFGE is the gold standard for strain genotyping also for *S. maltophilia*. PCR data shown in Table 3 partially complemented genotyping data obtained by PFGE. OBGTC9 and OBGTC10 strains exhibited the same PCR profile at all the SMAG-positive regions analyzed, and their PFGE profiles are undistinguishable (Fig. 3). Strains 916, 1019 and 1053, which belong to PFGE A-type, and strains 528 and 571, which belong to PFGE B-type (data not shown; for the PFGE relatedness of these strains, see Crispino *et al*., 2002) similarly exhibited the same PCR profile at all the *loci *(Table 3). Other strains exhibited similar PCR profiles, but different PFGE patterns. *Xba*I digests of OBGTC13, OBGTC23 and OBGTC30 DNAs produced undistinguishable PFGE patterns. Their PCR profiles were similar on the whole, but OBGTC23 featured a PCR type different from OBGTC13 and OBGTC30 (PT-10 vs PT-13), and the three isolates differed from each other at multiple additional *loci*. Moreover, K279a DNA was identical to OBGTC9 and OBGTC10 DNAs at all SMAG *loci*, but clearly differed from both DNAs when analyzed by PFGE (Fig. 3).

## Discussion

The MLVA technique involves amplification and size analysis of polymorphic DNA regions containing variable numbers of tandemly repeated sequences, and is an established method to classify isolates of microbial species for which complete genome information is available [22]. The determination of the complete genome sequence of the *S. maltophilia *K279a strain allowed us to set up a MLVA assay also for this organism.

All the approaches so far utilized for the genotyping of *S. maltophilia *clinical isolates have led to the conclusion that *S. maltophilia *strains are highly heterogeneous [5-11]. According to AFLP fingerprinting [5] and *gyr*B RFLP [6] analysis, *S. maltophilia *can be assigned to 8–10 genomic groups. The majority of CF isolates grouped in two clusters [6], suggesting that isolates of specific groups have an increased potential for the colonization of the respiratory tract of CF patients. Considering the interest paid to *S. maltophilia *as an emerging opportunistic pathogen associated with nosocomial infections, we wanted to develop a fast, accurate and unexpensive method of genotyping which could be adopted for strain classification and comparisons. The chromosomal regions carrying clusters of SMAGs, an abundant sequence repeat spread in the *S. maltophilia *genome, vary in size among isolates, allowing MLVA-based typing surveys. The repeat units found at the various *loci *analyzed are sufficiently large to discriminate length variation among isolates by low molecular weight agarose gel electrophoresis. In most instances, PCR data enabled to set a direct correlation between the length of the amplimers and the number of SMAG repeats present at the analyzed *loci*. The latter could be directly marked by the number of repeats, setting the basis for a simple, numerical classification of the strains analysed. Broad sorting of a large number of isolates may suggest to restrict MLVA analyses to a few SMAG-positive *loci*. Allelic variants of the four *loci *II, V, I and VII (Table 3) are detectable by two PCR co-amplifications reaction, providing 4-digit typing profiles which could turn out to be effective for simple typing purposes. By increasing the number of *loci *analyzed, it could be possible to obtain a more discriminating digit profile, as in MLST analyses [23].

According to our typing scheme, regions not responding to the PCR approach have been assigned the digit 0. While uninformative on the integrity of the region under scrutiny, the lack of amplification of a certain *locus *is fully exploitable in a multi-typing system. A "caveat" may be represented by cases in which it is not possible to immediately correlate the size of the amplicon with the number of repeats as observed for amplimers slightly differing in length occasionally found at *loci *VII, XI and XII. However, this could represent a problem in the analyses of large populations of isolates, calling for a highly discriminating profiling. Minor size differences among amplicons allow discrimination for typing purposes as the major ones, and can be indicated by marking amplimers also with letters. However, accurate measuring of small size differences among amplimers which have been analyzed in different electrophoretic runs may be cumbersome. Thus, it would be advisable to assign to amplicons which may slightly differ in size the same digit, which should correspond to the most likely number of repeats present. This may ensure to rapidly proceed in classifying the different isolates, eventually further distinguishing them by means of additional analyses.

MLVA assays provide results that parallel PFGE data, although some differences have been noticed, since MLVA and PFGE measure different types of chromosomal modifications, and, for example, recombination events within a genome could be detected by PFGE, but overlooked by MLVA analyses [18,24,25]. The same holds true in our study, as strains such as K279a, OBGTC9 and OBGTC10, while identical according to MLVA data, differ when analyzed by PFGE. The method we have devised is simpler, less time-consuming and economically more advantageous than PFGE. As suggested by Tenover *et al*. [25], MLVA approaches could be particularly helpful to identify strains responsible for outbreaks in hospital settings, and to determine the relatedness of isolates collected over short periods of time. In contrast, PFGE could be priviliged for long time period analyses of bacterial populations.

## Conclusion

The utilization of the present protocol will be useful for fast and efficient typing purposes. Several *S. maltophilia *isolates could be typed in hours, and the results interpreted *de visu *without the need for sophisticated software. Data would be easily reproducible, and immediately comparable among different laboratories.

## Methods

### *S. maltophilia *strains

*S. maltophilia *strains analyzed in this study are listed in Table 1. Clinical isolates were identified as *S. maltophilia *by using the VITEK II system (bioMerieux, Morey-l'Etoile, France). The identification was confirmed by PCR amplification and sequence analysis of the 16S rDNA. Strains were routinely grown in brain heart infusion at 37°C, except for the environmental strains LMG959, LMG10871, LMG10879 and OBGN1 which were grown at 30°C. In order to analyze the stability of the genomic regions under scrutiny, a few strains were sub-cultured in brain heart infusion at 37°C 5 times for 18–24 hrs.

### PCR amplification

The DNA of single colonies derived from the final subcultures was analyzed by PCR amplification of DNA regions of interest. Genomic DNA was extracted as described by De Gregorio *et al*. [26]. PCR reactions were carried out by incubating 20 ng of DNA with 160 ng of each primer in the presence of dXTPs (200 nanomoles), 1.5mM magnesium chloride and the Taq DNA polymerase Recombinant (Invitrogen). Because of the high GC content of the *S. maltophilia *genome (> 66%), all PCR reactions were carried out in GC-rich buffer (Roche). The oligomers used as primers, and the annealing temperatures, are listed in Table 2. Samples were incubated at 95°C for 5', and subsequently for 1' at 95°C, 1' at the annealing temperature and 1' at 72°C, for a total of 30 cycles. At the end of the cycle, samples were kept at 72°C for 7' before harvesting.

PCR products were electrophoresed on 1.5–2% agarose gels in 0.5×TBE buffer (45 mM Tris pH 8, 45 mM Borate, 0.5 mM EDTA) at 120 V (constant voltage). The 100 bp ladder (Fermentas) was used as molecular weight marker.

### PFGE analysis

Preparation of agarose plugs containing chromosomal DNA for PFGE analysis was performed using the PulseNet standardized procedure  The DNA plugs were digested with 60 U of *Xba*I (Roche Diagnostics) at 37°C fo 16 h. Genomic DNA fragments were separated by PFGE at 14°C on agarose 1.2% w/v gels in a clamped homogeneous field electrophoresis apparatus (CHEF-DRII system; Bio-Rad, Hemel Hempstead, UK), with pulse times ramped from 1 to 20 s over 21 h at 6.0 V/cm in 0.5× TBE. DNA fragments obtained from *Xba*I digestion of plugs containing chromosomal DNA of *Salmonella braenderup *strain H9B12 were used as molecular weight markers [27]**.**

Electrophoretic patterns were analyzed by UPGMA (Unweighted Pair Group Method with Arithmetic mean) using the Gel Compar II version 4.5 software (Applied Maths).

## Authors' contributions

ER and FR designed the PCR oligomers and carried out the DNA analyses, BC, MC and RF provided the *S. maltophilia strains *and performed the PFGE experiments, PPDN conceived the study and participated in its design and coordination, MSC and PPDN drafted the manuscript. All authors read and approved the final manuscript.
